# Effects of cannabidivarin (CBDV) on brain excitation and inhibition systems in adults with and without Autism Spectrum Disorder (ASD): a single dose trial during magnetic resonance spectroscopy

**DOI:** 10.1038/s41398-019-0654-8

**Published:** 2019-11-20

**Authors:** Charlotte M Pretzsch, Bogdan Voinescu, David Lythgoe, Jamie Horder, Maria Andreina Mendez, Robert Wichers, Laura Ajram, Glynis Ivin, Martin Heasman, Richard A. E. Edden, Steven Williams, Declan G. M. Murphy, Eileen Daly, Gráinne M. McAlonan

**Affiliations:** 1 Department of Forensic and Neurodevelopmental Sciences, Institute of Psychiatry, Psychology & Neuroscience, King’s College London, London, UK; 2 Department of Neuroimaging Sciences, Institute of Psychiatry, Psychology & Neuroscience, King’s College London, London, UK; 3 South London and Maudsley NHS Foundation Trust Pharmacy, London, UK; 4 Russel H Morgan Department of Radiology and Radiological Science, Johns Hopkins Medical Institutions, Baltimore, MD USA

**Keywords:** Predictive markers, Autism spectrum disorders, Molecular neuroscience, Clinical pharmacology

## Abstract

Autism spectrum disorder (ASD) is a high cost neurodevelopmental condition; and there are currently no effective pharmacological treatments for its core symptoms. This has led some families and researchers to trial alternative remedies – including the non-intoxicating Cannabis sativa-derived compound cannabidivarin (CBDV). However, how CBDV affects the human brain is unknown. Previous (pre)clinical evidence suggests that CBDV may modulate brain excitatory-inhibitory systems, which are implicated in ASD. Hence, our main aim was to test, for the first time, if CBDV shifts glutamate and/or GABA metabolites – markers of the brain’s primary excitatory and inhibitory system - in both the ‘typical’ and autistic brain. Our subsidiary aim was to determine whether, within ASD, brain responsivity to CBDV challenge is related to baseline biological phenotype. We tested this using a repeated-measures, double-blind, randomized-order, cross-over design. We used magnetic resonance spectroscopy (MRS) to compare glutamate (Glx = glutamate + glutamine) and GABA + (GABA + macromolecules) levels following placebo (baseline) and 600 mg CBDV in 34 healthy men with (*n* = 17) and without (*n* = 17) ASD. Data acquisition from regions previously reliably linked to ASD (dorsomedial prefrontal cortex, DMPFC; left basal ganglia, BG) commenced 2 h (peak plasma levels) after placebo/CBDV administration. Where CBDV significantly shifted metabolite levels, we examined the relationship of this change with baseline metabolite levels. Test sessions were at least 13 days apart to ensure CBDV wash-out. CBDV significantly increased Glx in the BG of both groups. However, this impact was not uniform across individuals. In the ASD group, and not in the typically developing controls, the ‘shift’ in Glx correlated negatively with baseline Glx concentration. In contrast, CBDV had no significant impact on Glx in the DMPFC, or on GABA+ in either voxel in either group. Our findings suggest that, as measured by MRS, CBDV modulates the glutamate-GABA system in the BG but not in frontal regions. Moreover, there is individual variation in response depending on baseline biochemistry. Future studies should examine the effect of CBDV on behaviour and if the response to an acute dose of CBDV could predict a potential clinical treatment response in ASD.

## Introduction

Autism spectrum disorder (ASD) is a complex neurodevelopmental condition estimated to affect up to 1 in 59 individuals^1^. ASD incurs a high cost. For instance, the average life expectancy in individuals with high functioning ASD is 12 years shorter than that in so-called ‘neurotypicals’^2^. Despite this, there are no effective pharmacological treatments for the core symptoms of ASD. Researchers are therefore increasingly exploring alternative remedies, such as the non-intoxicating *Cannabis sativa-*derived compounds cannabidiol (CBD) and cannabidivarin (CBDV)^3,4^. For instance, in a recently published study using magnetic resonance spectroscopy (MRS) we examined, for the first time, the effects of CBD on brain excitatory (E) glutamate and inhibitory (I) γ-aminobutyric acid (GABA) – given that E-I abnormalities may be one of the key mechanisms underpinning ASD^3^. We found that CBD modulated glutamate-GABA systems in both ASD and neurotypicals, but that prefrontal GABA systems responded differently in ASD. Specifically, CBD decreased prefrontal GABA levels in ASD but not in controls. These findings contributed to increasing evidence that an important effect of therapeutic compounds derived from *Cannabis sativa* may be the ‘shifting’ of brain E-I systems.

There is a growing number of clinical trials of these compounds, including the first trial in ASD, which investigates the impact of CBDV on irritability in autistic children (clinicaltrials.gov, identifier: NCT03202303). Unfortunately, however, most clinical trials which address ‘core’ social and/or repetitive behaviour symptoms of ASD fail. There are many reasons for this – but principal among them are that the candidate treatment may not ‘shift’ key mechanisms implicated in the disorder, and/or that they use an ‘all-comers’ approach (i.e., all patients meeting a broad set of eligibility criteria are included)^5^. This is relevant to ASD, which is a highly heterogeneous condition, where the response to candidate treatments may vary between individuals and where no single approach is likely to succeed for everyone^5^. A pressing goal should therefore be to determine objectively how candidate treatments, such as CBDV, impact upon the typical and atypical (autistic) brain; and who may be (biologically) responsive prior to clinical trial. Establishing an objective marker of biological response or ‘target engagement’ (e.g., using a marker of brain biochemistry) may be useful when a change in a biological target is linked to a change in behaviour. This may improve the success of drug trials by helping to predict if someone will respond – or not – to a pharmacological treatment with a change in behaviour, i.e., it may inform stratification approaches. This has not yet been achieved for CBDV.

We know from preclinical studies that CBDV may act on several neuroglial targets. Many of these effects are thought to converge to modulate glutamatergic and GABAergic pathways. For instance, CBDV binds to several members of the Transient Receptor Potential (TRP) family, including vanilloid type 1 (TRPV1), vanilloid type 2 (TRPV2), and ankyrin type 1 (TRPA1) receptors^6^ and, upon binding, CBDV is thought to activate and rapidly desensitize these receptors. TRP receptors are located on cortical and subcortical excitatory pyramidal cells^7^, inhibitory interneurons^7^, and microglia^8^, including in the basal ganglia (BG) and the dorsomedial prefrontal cortex (DMPFC)^9–13^. Thus, CBDV could potentially modulate the activity of neurons and glia directly involved in E-I regulation^6^ in key cortical and subcortical neural hubs. This is especially relevant to ASD, where subtle E-I alterations in several cortical-subcortical circuits^14–16^, including in the BG and DMPFC, have been linked to ASD symptoms^17,18^. Despite this, no study to date has examined the effect of CBDV on cortical and/or subcortical glutamate and/or GABA indices in-vivo in neurotypical individuals, let alone those with ASD.

Previous evidence suggests that autistic individuals may respond atypically to pharmacological challenge. For instance, in our recent study we discovered that, in the BG and DMPFC, CBD elicited a similar response in glutamate systems, but an opposite effect in GABA systems in the neurotypical compared to the autistic adult brain^3^. In addition to this between-group difference, our work has also revealed that there is response variability to pharmacological challenge within the autistic population^3,18^. These findings raise the question as to whether the biological response to CBDV also varies within the autistic population; and if so, which phenotypic measures may help predict treatment response. However, whether biology helps predict a (drug-induced) shift in E-I metabolite concentrations in ASD remains to be investigated.

Therefore, we examined for the first time: (1) the biological impact of CBDV on the typical and autistic brain, (2) if the response to CBDV is uniform across groups, and (3) the potential biological (baseline) correlates of response variability within ASD.

We tested this using a repeated-measures, randomized-order, placebo-controlled, double-blind, cross-over design. We compared magnetic resonance spectroscopy (MRS) measures of Glx (glutamate + glutamine) and GABA + (GABA+ macromolecules, see Methods) in adult men with and without a diagnosis of ASD following a single oral dose of 600 mg CBDV or a matched placebo. Data were collected from the BG and DMPFC because these regions have been reliably implicated in ASD, and alterations in E-I dynamics can be detected in these targets using MRS^3,13–18^. To explore variability of the drug response (where CBDV significantly shifted metabolite levels), we examined the relationship of any CBDV induced shift in metabolite with baseline metabolite levels.

## Materials and Methods

### Procedure

This research was conducted in accordance with the Declaration of Helsinki, at the Institute of Psychiatry, Psychology, and Neuroscience (IoPPN) at De Crespigny Park, SE5 8AF, London, UK (August 2016 to August 2018). All our participants provided written informed consent. Every participant took part in all aspects of this case-control, placebo-controlled, randomised, double-blind, repeated-measures, cross-over study. This research was conducted as part of a larger study into the role of phytocannabinoids in ASD (ethical approval provided by the King’s College London Research Ethics Committee, reference HR15/162744). Although the UK Medicines and Health Regulatory Authority (MHRA) confirmed that our experimental design was not a Clinical Trial, for transparency the study was registered on clinicaltrials.gov (identifier: NCT03537950, entry name: HR15-162744).

Drugs (placebo, PLC; or CBDV) were allocated in a pseudo-randomised order so that approximately half in each group attended a placebo visit before CBDV; and half attended a CBDV visit before placebo. This randomisation was conducted by Prof. McAlonan using a random number generator (https://www.random.org/). All participants and researchers directing the study were blinded to the order of drug/placebo exposure. Participants attended for two visits, which were separated by a minimum of 13 days to allow for drug wash-out. On each visit, we collected urine samples to screen for illicit substances (a full list is included below). Subsequently, participants underwent a brief health check, received a liquid oral dose of the pharmacological probe (600 mg of CBDV; in line with previous single dose studies of non-psychoactive cannabinoids in adults (e.g., ref. ^19^) or a matched placebo, both provided by GW Research Ltd, Cambridge, UK), and then underwent a second brief health check to test for potential acute adverse reactions/side effects. Participants commenced scanning timed to coincide with peak plasma (2 h) concentration. After the scan, participants underwent a final health check to ensure they had experienced no ill-effects and were fit to leave the department. Participants experienced no ill/unintended effects.

### Participants

We excluded all potential participants that had a comorbid major psychiatric or medical disorder affecting brain development (e.g. schizophrenia or epilepsy), a known genetic condition associated with ASD (e.g. Fragile X syndrome), a history of brain/head injury, a full-scale intelligence quotient (FSIQ) below 70, or who were reliant on receiving regular medication known to directly influence Glx or GABA levels+, such as benzodiazepines. Participants were asked to refrain from using cannabis and/or other illicit substances in the month before scanning, and from drinking alcohol on the day before testing. Data from all individuals that screened positive for illicit substances in the urine drug screening were excluded. As a result, we retained data from 34 subjects (17 neurotypicals, 17 individuals with ASD) (see Table 1 for demographics). Power analyses suggested that this sample size was enough to detect a drug-induced 10% change in Glx (e.g. after ketamine administration^20^) at a power of 0.8 and a significance level of α = 0.05. All autistic participants had a clinical diagnosis of ASD according to ICD10 research criteria^21–23^, and their severity of symptoms was confirmed using standardised research diagnostic instruments where appropriate (*ADOS* Autism Diagnostic Observation Schedule^23^; and *ADI-R* Autism Diagnostic Interview-Revised^22^).

**Table 1:** Participant demographics Summary of participant demographics for all subjects, unless otherwise specified.

Variable (SD)	TD	ASD	F(Dof)	*P*-value
N (M/F)	17 (17/0)	17 (17/0)		
Age (Y)	28.47 (6.55)	31.29 (9.94)	F(1) = 0.956	0.335
Time between visits	28.15 (15.19)	32.43 (16.54)	F(1) = 0.487	0.492
FSIQ	124.59 (12.7)	111.35 (18.80)	F(1) = 5.781	**0.022**
ADI COM		8.10 (6.77) (*n* = 10)		
ADI SOC		9.30 (4.88) (*n* = 10)		
ADI RRSB		3.60 (2.17) (*n* = 10)		
ADOS COM		4.53 (2.79) (*n* = 17)		
ADOS IM		1.07 (0.80) (*n* = 15)		
ADOS SBRI		1.50 (1.51) (*n* = 16)		
ADOS SOC		7.94 (3.77) (*n* = 17)		

*ADI* Autism diagnostic interview, (*COM* communication subscale, *SOC* social interaction subscale, *RRSB* restricted, repetitive, and stereotyped patterns of behavior subscale), *ADOS* Autism diagnostic observation schedule (*COM* communication subscale, *IM* imagination and creativity subscale, *SOC* reciprocal social interaction subscale, *SBRI* stereotyped behaviors and restricted interests subscale), *ASD* Autism spectrum disorder, *F(Dof)* F-value and degrees of freedom, *F* Female, *FSIQ* Full-scale intelligence quotient, *M* Male, *N* Number of subjects, *SD* Standard deviation, *TD* Typically developing controls, *y* years

### Imaging data acquisition

We acquired all our imaging data on a 3 T GE Excite II magnetic resonance imaging (MRI) scanner (GE Medical Systems, Milwaukee, WI, USA). Our scanning protocol included a structural MRI scan acquired using a 3D inversion recovery prepared fast spoiled gradient recalled (IR-FSPGR) sequence (slice thickness = 1.1 mm, spatial positions = 124, flip angle = 20°, field of view (FoV) = 280 mm, echo time (TE) = 2.844 ms, repetition time (TR) = 7.068 ms, inversion time = 450 ms, matrix = 256 × 256). This structural scan was conducted to obtain information used during the preprocessing of the spectroscopy scan. The scanning protocol further included a spectroscopy scan based on the MEshcher-GArwood Point RESolved Spectroscopy (MEGA-PRESS) sequence. We acquired data (352 datapoints) from two voxels: the first was positioned in the BG (echo time (TE) = 68 ms, repetition time (TR) = 1800 ms, voxel size = 35 × 30 × 25 mm^3^). The second voxel was positioned in the DMPFC (TE = 68 ms, TR = 2000 ms, voxel size = 25 × 40 × 30 mm^3^).

### Urine test

We performed liquid chromatography–mass spectrometry (LC–MS) analysis on urine samples provided by each subject before the drug administration to evaluate presence or absence of illicit substances that could confound our results. Therefore, all participants that screened positive for any of the drugs tested, including Amphetamines (Amphetamine, Methamphetamine, MDMA/Ecstasy), Benzodiazepines, Cannabis, Cocaine (as benzoylecgonine), Methadone and its metabolite EDDP, and Opioids (6-monoacetylmorphine, morphine, codeine, dihydrocodeine) were excluded from further analysis. This led to the exclusion of four subjects (two typically developing controls, two ASD) from the original sample.

### Data processing

#### Structural data processing

We inspected all T1-weighted structural MRI volumes manually to ensure adequate signal-to-noise ratio (SNR) and the absence of motion artefacts. Subsequently, we normalised our structural volumes to Montreal Neurological Institute (MNI) space and segmented them into grey matter (GM), white matter (WM), and cerebrospinal fluid (CSF), to obtain percentage measures of tissue composition in each individual MRS voxel, using positional coordinates embedded in the raw spectra data files.

### Magnetic resonance spectroscopy data processing

We pre-processed our MRS data using in-house scripts adapted from FID-A^24^, which prepared the data for reading into the main processing software. This pre-processing comprised converting data to the correct file format, combining receiver channels, removing ‘bad’ averages (>4 standard deviations), correcting frequency drift (alignment of averages), separating and visualizing the edit on/off spectra, and subtracting them to produce the difference spectrum. We further inspected all spectra manually to ensure adequate SNR as well as the absence of artefacts^25,27^.

We processed our MRS data using LCModel v6.3-1L software (Stephen Provencher Incorporated, Oakville, Canada). This software uses a linear combination of model spectra derived from metabolite solutions in vitro to analyse the major resonances of in vivo spectra. Here, we used a basis set (mega-press-3T-1) to determine concentrations of GABA + (40% of the GABA signal represents macromolecules)^25^ and Glx (glutamate + glutamine). The spectra also included glutathione (GSH), N-acetyl-aspartate (NAA), N-acetyl-aspartylglutamate (NAAG), NAA + NAAG, and GSH + Glu + Gln in each voxel; however, for the purposes of this study, we focused only on GABA+ and Glx.

Partial volume effects (different proportions of GM, WM, and CSF in the MRS voxels) are a potential confound in MRS, especially in light of previously reported volumetric differences between autistic and neurotypical individuals^26^. Therefore, to account for partial volume effects, we corrected all metabolite values for GM, WM, and CSF percentages. Assuming that CSF only contains negligible quantities of the metabolites of interest, the calculations were as follows: LCModel assumes a voxel consists to 100% of WM with a water concentration (WCONC) of 35880 mM and corrects each metabolite value (where F stands for fraction) using the factor: (43300 ∗ F_GM_ + 35880 ∗ F_WM_ + 55556 ∗ F_CSF_)/(1 − F_CSF_). To correct for the value of water concentration being used in the processing through LCModel, we divided values by an individual correction factor (35880), arriving at (1.207 ∗ F_GM_ + F_WM_ + 1.548 ∗ F_CSF_)/(1 − F_CSF_). Thus, our corrected metabolite values were obtained by multiplying the raw metabolite values by this correction. Since we did not measure relaxation times for tissue water and metabolites, these were not corrected for - with the exception of assuming the tissue water relaxation time (T_2_ = 80 ms)^27^.

Moreover, to improve the robustness of our findings, we excluded all measurements of Glx (glutamate + glutamine) and GABA+ where the Cramér-Rao lower bound (CRLB) estimates exceeded 15% from further analysis (LCModel manual, Stephen Provencher Incorporated, Oakville, Canada). Based on this, we excluded eight data points from three subjects (1 neurotypical, 2 ASD): neurotypicals: 1: DMPFC GABA+_CBDV_; ASD: 2: BG Glx_PLC_; 3: DMPFC GABA+_PLC_ & Glx_PLC_, DMPFC GABA+_CBDV_ & Glx_CBDV_, and BG GABA+_CBDV_ & Glx_CBDV_.

### Statistical analysis

We compared demographic measures (age, FSIQ) and baseline levels of Glx and GABA+ in each region of interest using a one-way ANOVA (significance level *p* < 0.05).

To examine our primary hypothesis that CBDV shifts Glx and/or GABA+ in our two brain regions of interest (BG and DMPFC), we conducted 2 × 2 mixed-model ANOVAs with group (neurotypicals, ASD) as the between-subject factor, and drug (PLC, CBDV) as the within-subject factor for each metabolite in each region separately. We included the following subject numbers: for Glx measures in BG, ASD *n* = 12, neurotypicals *n* = 11; for Glx measures in DMPFC, ASD *n* = 12, neurotypicals *n* = 13; for GABA+ measures in BG, ASD *n* = 12, neurotypicals *n* = 13; and for GABA+ measures in the DMPFC, ASD *n* = 11, neurotypicals *n* = 11. With the caveat that Bonferroni correction can be overly conservative, we also report Bonferroni corrected *p*-values alongside any significant uncorrected results.

We examined the relationship between significant drug-induced shifts in metabolite levels (CBDV-PLC) and baseline metabolite measures across the ASD group using Pearson’s correlation analyses (*p* < 0.05).

Analyses were carried out using SPSS 24.00 software (SPSS, Chicago, IL, USA), and graphs were generated using GraphPad Prism version 7 for Mac, GraphPad Software, La Jolla, CA, USA, www.graphpad.com.

## Results

### Demographics

We retained data from 17 neurotypicals (three of those only had a scan after placebo and not after CBDV), and from 17 individuals with ASD (four of those only had a scan after placebo and not after CBDV). Groups were similar in age (F(1) = 0.956, *p* = 0.335), but, as is commonly reported, autistic individuals had a slightly lower FSIQ than typically developing controls and this difference was significant (F(1) = 5.781, *p* = 0.022). However, FSIQ did not correlate with drug-induced metabolite shifts across groups (all *r* ≤ 0.34, all *p* ≥ 0.122). FSIQ correlated with Glx shift in the BG within the ASD group (*r* = 0.70, *p* = 0.016, *n* = 11), but this correlation was not significant in the neurotypicals (*r* = −0.10, *p* = 0.765, *n* = 11) and did not differ significantly between groups (*p* > 0.05). This suggests that the between-group difference in FSIQ did not influence our results (Table 1).

### Tissue composition and data quality

Tissue percentages (not excluding omitted spectra) differed significantly between groups for BG PLC GM (F(1) = 7.307, *p* = 0.011), BG PLC WM (F(1) = 9.345, *p* = 0.004), BG CBDV GM (F(1) = 6.70, *p* = 0.016), and for BG CBDV WM (F(1) = 7.89, *p* = 0.010); but not for other tissues, as summarised in Table 2. This is in line with previous studies that have demonstrated morphological differences in the BG in autistic compared to neurotypical individuals^26,28^. Moreover, as the composition properties of these tissues differed significantly at baseline and during the drug condition, this difference was not induced through CBDV. In the statistical analysis we corrected all metabolite values accordingly.

**Table 2:** Percentage of GM, WM, and CSF in voxels of interest Absolute values (and standard deviations).

Voxel	Drug	Tissue	TD	ASD	F(dof)	*p*-value
**BG**	**PLC**	GM (SD)	42.53% (3.03%)	45.43% (3.21%)	F(1) = 7.307	**0.011**
		WM (SD)	50.47% (3.39%)	46.54% (4.08%)	F(1) = 9.345	0.004
		CSF (SD)	6.92% (1.33%)	7.95% (1.95%)	F(1) = 3.222	0.082
	**CBDV**	GM (SD)	41.65% (2.40%)	44.68% (3.47%)	F(1) = 6.70	**0.016**
		WM (SD)	51.71% (2.95%)	47.23% (4.94%)	F(1) = 7.89	**0.010**
		CSF (SD)	6.56% (1.36%)	8.01% (2.48%)	F(1) = 3.42	0.077
**DMPFC**	**PLC**	GM (SD)	52.93% (2.21%)	52.38% (3.49%)	F(1) = 0.299	0.589
		WM (SD)	27.24% (3.36%)	28.11% (3.65%)	F(1) = 0.527	0.473
		CSF (SD)	19.73% (4.05%)	19.41% (2.57%)	F(1) = 0.076	0.784
	**CBDV**	GM (SD)	52.68% (3.16%)	53.41% (3.67%)	F(1) = 0.30	0.590
		WM (SD)	27.94% (4.23%)	27.52% (4.13%)	F(1) = 0.07	0.802
		CSF (SD)	19.25% (5.13%)	18.97% (2.22%)	F(1) = 0.03	0.857

*ASD* Autism spectrum disorder, *BG* Basal ganglia, *CBDV* Cannabidivarin, *CSF* Cerebrospinal fluid, *DMPFC* Dorsomedial prefrontal cortex, *F(dof)* F-value and degrees of freedom, *GM* Grey matter, *PLC* Placebo, *SD* Standard deviation, *TD* Typically developing controls, *WM* White matter

To ensure that the MRS data quality did not differ between groups, we compared the LCModel Cramér-Rao Lower Bound estimates for each metabolite (Glx, GABA+) in each voxel (excluding omitted spectra), using a one-way ANOVA^29^. As predicted, there were no significant differences (all F(1) ≤ 2.751, all *p* ≥ 0.107), as depicted in Table 3.

**Table 3:** Cramér-Rao Lower Bound estimates for each metabolite (Glx, GABA+) in each voxel Absolute values (and standard deviations).

Voxel	Drug	Metabolite	TD	ASD	F(dof)	*p*-value
**BG**	**PLC**	GABA+	4.56 (0.66)	4.82 (0.81)	F(1) = 1.095	0.303
		Glx	7.50 (2.09)	7.56 (2.25)	F(1) = 0.007	0.935
	**CBDV**	GABA+	4.38 (0.51)	4.79 (0.80)	F(1) = 2.371	0.136
		Glx	6.77 (1.36)	7.21 (2.29)	F(1) = 0.368	0.549
**DMPFC**	**PLC**	GABA+	6.47 (0.87)	7.38 (2.06)	F(1) = 2.751	0.107
		Glx	5.68 (0.73)	5.88 (1.02)	F(1) = 0.416	0.524
	**CBDV**	GABA+	6.50 (0.67)	7.07 (1.73)	F(1) = 1.153	0.294
		Glx	5.85 (1.14)	5.57 (0.94)	F(1) = 0.469	0.500

*ASD* Autism spectrum disorder, *BG* Basal ganglia, *CBDV* Cannabidivarin, *CSF* Cerebrospinal fluid, *DMPFC* Dorsomedial prefrontal cortex, *F(dof)* F-value and degrees of freedom, *GABA+* gamma-aminobutyric acid + macromolecules, *Glx* glutamate + glutamine, *PLC* Placebo, *SD* Standard deviation, *TD* Typically developing controls

Extended MRS studies can be confounded by ‘drift’, where metabolite estimates on the same scanner change across long periods of time. Therefore, we compared the inter-scan duration (days between PLC and CBDV scan) between groups; and found no significant difference (F(1) = 0.487, *p* = 0.492), as shown in Table 1. Moreover, scan date for each drug condition (PLC, CBDV) did not correlate with metabolite values at that drug condition (all Pearson’s *r* ≤ 0.329, all *p* ≥ 0.087), confirming that data acquisition was stable over time.

### Metabolite differences

#### Glx (glutamate + glutamine)

**Fig. 1:**
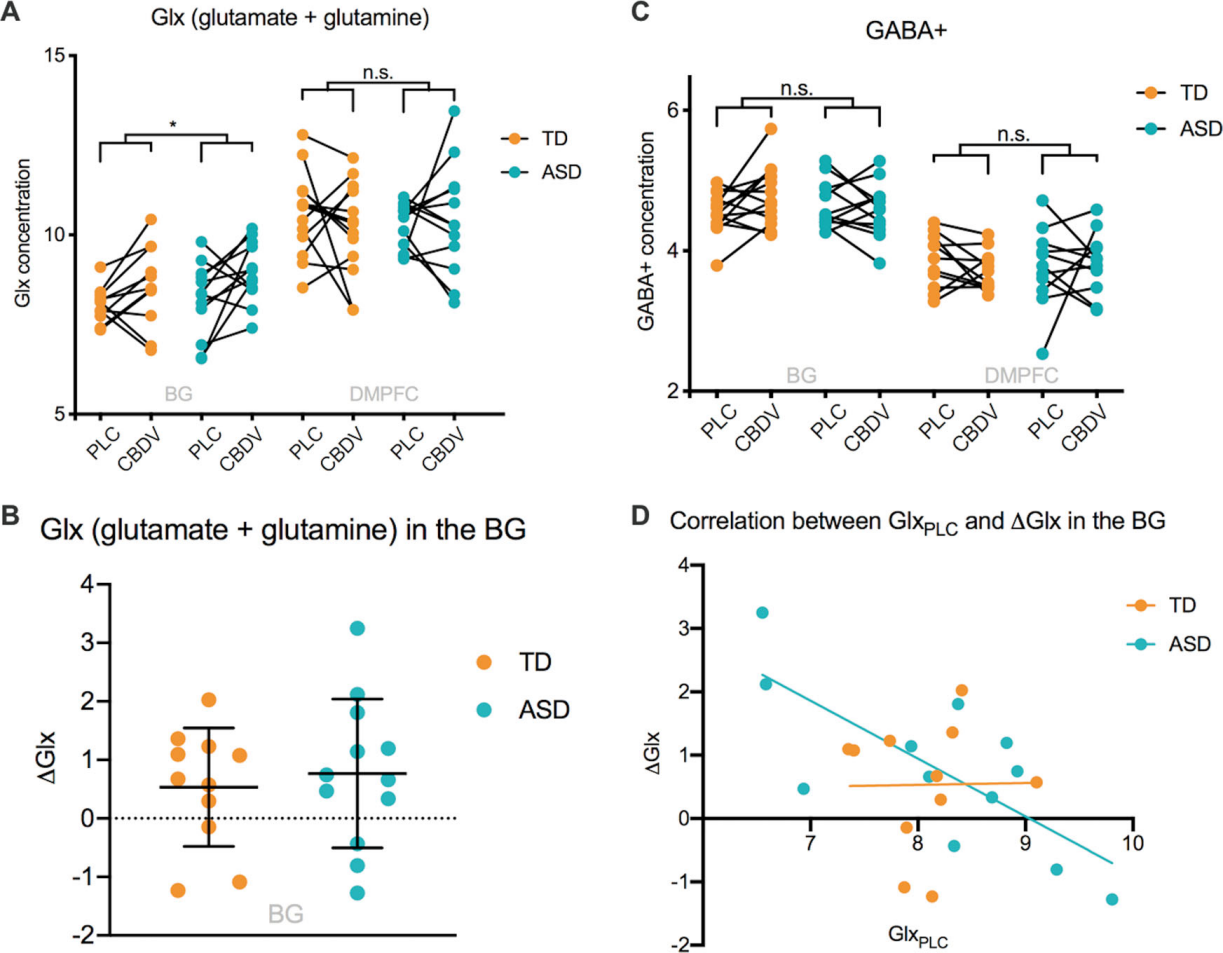
**a** Glx (glutamate + glutamine) in the basal ganglia and dorsomedial prefrontal cortex for both groups in both drug conditions. Glx concentration (y-axis) represents the ratio of the Glx metabolite resonance area to the unsuppressed water resonance area. **b** Drug-induced shift in Glx in the basal ganglia. ΔGlx (y-axis) represents the cannabidivarin (CBDV)-induced shift in Glx concentration compared to placebo (PLC), i.e. CBDV-PLC. Group means are indicated by black horizonal bars. Error bars represent standard deviations. **c** gamma-aminobutyric acid + macromolecules (GABA+) in the basal ganglia and dorsomedial prefrontal cortex for both groups in both drug conditions. GABA+ concentration (y-axis) represents the ratio of the GABA+ metabolite resonance area to the unsuppressed water resonance area. **d** Correlation between Glx at baseline and CBDV-induced shift in Glx (ΔGlx) in the basal ganglia. *ASD* Autism spectrum disorder, *BG* Basal ganglia, *CBDV* Cannabidivarin, *DMPFC* Dorsomedial prefrontal cortex, *Glx* glutamate + glutamine, *ns* Not significant, *PLC* Placebo, *TD* Typically developing controls, * indicates a significance level at *p* ≤ 0.05.

In the BG, we observed a significant effect of drug (F(1, 21) = 7.268, p_uncorr_ = 0.014, η^2^ = 0.257). Across groups, CBDV increased Glx compared to PLC, as displayed in Fig. 1a, b. This effect survived correction for multiple comparisons across voxels (p_corr_ = 0.03). In contrast, there was no significant effect of group (F(1) = 0.763, *p* = 0.392, η^2 ^= 0.035), and no group × drug interaction effect (F(1, 21) = 0.235, *p* = 0.633, η^2 ^= 0.011). In the DMPFC, there was no significant effect of drug or group, and no group × drug interaction effect (Fig. 1a).

#### GABA+

We observed no significant main or interaction effects within either voxel (Fig. 1c).

### Relationship to baseline metabolite measures

There was a significant negative correlation between the CBDV-induced BG Glx shift and baseline Glx levels in ASD (*r* = −0.749, *p* = 0.005, *n* = 12), but not in the typically developing controls (*r* = 0.014, *p* = 0.967, *n* = 11). This correlation differed significantly between groups (*p* = 0.04). (Fig. 1d). In interpreting this result, we caution that recent studies suggest that correlations between baseline and change scores in randomized controlled trials are common and may represent a regression to the mean, rather than the treatment effect^30^. To account for this effect in our analyses, we re-ran our repeated-measures ANOVAs as ANCOVAs with baseline metabolite levels as a covariate and found that the directionality and significance of our results remained the same. We still observed an opposite direction of correlation between baseline levels and drug-induced changes in Glx in the two groups. We emphasize that our study is not designed to investigate the degree to which any correlation with baseline reflects underlying neurobiology or a purely statistical effect. However, it is possible that a biological group differences at least partly contributes to the different relationships found in each group.

## Discussion

We have demonstrated that a single acute dose of CBDV ‘shifts’ subcortical levels of Glx, the brain’s primary excitatory neurotransmitter, in the living adult human brain. The direction of shift was the same across the neurotypical and autistic participants, i.e., CBDV induced a mean increase in BG Glx in both groups. However, the response to CBDV varied within the ASD group and correlated negatively with baseline BG Glx levels. In contrast, CBDV had no impact on Glx in DMPFC, nor on GABA+ levels in either voxel. Moreover, congruent with some^18,30^, but not all previous MRS studies of glutamate and GABA in ASD^18,31,32^ in the BG and DMPFC, there were no between-group differences in baseline metabolite levels.

Thus, our study suggests that CBDV targets subcortical excitatory glutamate systems both in autistic and neurotypical adults; but that individual responses in autistic brains vary depending on baseline Glx levels.

### Neurobiological underpinnings of the effect of CBDV on Glx in the BG

Our findings suggest that CBDV may serve as a tool to shift Glx in specific subcortical regions (the left BG) both in the neurotypical and autistic brain. The neurobiological underpinnings of this effect, however, are not entirely clear. Previous evidence suggests several possible neurobiological mechanisms through which CBDV may influence Glx pathways in the BG. For instance, preclinical studies have shown that the BG contain a wealth of excitatory pyramidal (and inhibitory projection) neurons^33^, which are densely surrounded by TRP receptors (including TRPV1, TRPV2, and TRPA1)^10,34,35^. CBDV may have bound to and subsequently activated these pyramidal neuron-bound TRP receptors, thereby increasing Glx in this region. This is supported by previous evidence that CBDV may modulate excitatory neurotransmission through a TRP receptor-dependent mechanism^6^. The BG also contain a particularly high number of microglia^36^; and it is possible that CBDV increased Glx levels here by activating microglial TRP receptors. Specifically, activation of TRP receptors has been shown to upregulate microglial activity and migration^8^, which is known to enhance extracellular vesicular shedding and subsequent glutamate release^8,37^. The prefrontal cortex has a different configuration of receptors and cells compared to the BG, which might explain a different response to CBDV here. We have previously reported that CBD causes a differential effect on prefrontal GABA+ in the autistic compared to neurotypical brain. Unfortunately, the cellular mechanisms underpinning the effects of cannabinoids have not yet been fully uncovered. Consequently, we do not yet know which cellular response differences contribute to their divergent effects on excitatory and inhibitory metabolites at ‘bulk tissue’ level. In sum, across groups, CBDV may have shifted Glx through multiple neuroglial mechanisms.

### Drug-response variability in ASD in relation to baseline measures

Although the mean effect of CBDV on Glx was uniform across groups, the drug response varied within groups. In ASD (but not in the neurotypicals), the drug-induced shift in BG Glx correlated significantly (negatively) with baseline Glx. Specifically, in the autistic individuals with the lowest baseline Glx measures, CBDV increased Glx levels; whereas the individuals with the highest baseline Glx measures experienced a decrease in Glx. Thus, these findings highlight the presence of intra-group response variability within ASD, where subjects may respond to drug challenge not just to different extents but also in opposite directions. Consequently, clinical trials of CBDV may find that not every participant responds similarly to CBDV treatment. Further research efforts may need to focus on identifying participant subgroups that will respond to treatment in a similar way.

### Implications of a BG Glx shift for cognition and behaviour

The utility of this observed shift in BG Glx for predicting an individual’s long term (clinical) treatment response is unclear; and was not part of our study. Previous research suggests that the BG are connected with other cortical and subcortical regions in the form of subcortical-cortical-thalamic loops, which are regulated and maintained by excitatory and inhibitory neurotransmission^38,39^. These loops support a range of cognitive functions and behaviours, such as reward, learning, memory, and motor processing^39–50^, which can be impaired in ASD^51,52^. Future studies should therefore examine the impact of CBDV on these cognitive processes and behaviours to determine if CBDV may offer clinical benefits in specific autistic subgroups.

### Limitations

Our findings must be considered alongside several limitations. We measured metabolite concentrations using MRS, which is unable to distinguish between the specific metabolite contributions to both the Glx and GABA+ signal. Moreover, the poor spatial resolution of MRS only allows ‘bulk’ assessments, which limited our ability to discern intra- and extra-cellular metabolite levels. Additional studies using more advanced technologies, such as (ultra-) high-field MRS are required to address this limitation.

MRS further has the disadvantage of relying on pre-selected regions-of-interest to be defined before the study. Thus, we cannot know how CBDV might modulate metabolites in other parts of the brain. This may change in the future with the development of sequences that allow whole-brain scanning^53^. At this point however, we are not aware of a scanning sequence that allows whole-brain spectroscopy of glutamate and GABA in a reasonable time frame.

Also, our sample size in this pilot study was modest. This was due to (i) our strict inclusion and exclusion criteria (e.g. exclusion of participants using glutamate and GABA-acting drugs); (ii) our time-intensive repeated-measures testing (involving drug administration), which was not always practical for participants; and (iii) our rigorous data quality control, e.g. exclusion of datasets based on head motion, which is known to be an issue in ASD. However, our repeated-measures design mitigated against this by reducing inter-subject variability (each subject acted as their own ‘control’) and thus increasing statistical power. Also, our sample size was comparable to (or bigger than) that in previous MRS studies in ASD^18,30^.

Moreover, CBDV remains an under-investigated compound. For instance, although plasma levels of CBDV are thought to peak on average two hours after administration, the exact times and concentrations may vary between individuals depending on several factors such as age, body-weight, size, genetics, absorption, distribution, and metabolism^54^. This made capturing the maximum drug effects for each participant challenging. Therefore, to minimize variability in drug levels, all efforts were made to match participants (e.g. by age) and to keep study procedures and timings consistent. Nonetheless, future studies should explore in more detail the pharmacodynamics of CBDV (e.g. effects of acute vs steady-state/long-term dosing), origins of inter-subject variability, and how this can be accounted for in pharmacological studies.

Finally, this was a first study of potential tissue level changes in metabolites following CBDV. Achieving even this modest sample size required a total of around 76 study visits. Therefore, we prioritized sample homogeneity and applied strict exclusion criteria. However, this approach has the disadvantage of potentially limiting the generalizability of our findings. We hope that having observed a biological response to CBDV in adult men with and without ASD (i.e. a Proof of Concept) will encourage subsequent studies to explore the effects of CBDV in women, children, those with intellectual difficulties, and other groups.

## Conclusions

Here we report that CBDV can ‘shift’ subcortical levels of the brain’s primary excitatory metabolite glutamate (measured as Glx) *both* in the neurotypical and autistic brain; but that there may be significant response variability in ASD. These findings add to our understanding of the effects of CBDV in the adult human brain. Nonetheless, future studies will need to explore (i) the mechanisms of action of CBDV; (ii) the impact of CBDV on (ASD-related) cognition and behaviour; (iii) if single-dose responsivity could facilitate the identification of pharmacologically homogeneous sub-groups; and (iv) if acute CBDV effects are indicative of the impact of long-term treatment in ASD.
